# Neuro-Fuzzy Network-Based Nonlinear Hybrid Active Noise Control Systems

**DOI:** 10.3390/e27020138

**Published:** 2025-01-28

**Authors:** Thi Trung Tin Nguyen, Jing Na, Le Thai Nguyen, Xian Wang

**Affiliations:** 1 Faculty of Mechanical & Electrical Engineering, Kunming University of Science & Technology, Kunming 650500, China; 20201103008@stu.kust.edu.cn (T.T.T.N.); najing25@kust.edu.cn (J.N.); 2 Faculty of Engineering and Technology, Nguyen Tat Thanh University, Ho Chi Minh City 700000, Vietnam; nlthai@ntt.edu.vn

**Keywords:** active noise control, neural network, nonlinear filter, hybrid active noise control

## Abstract

Active noise control (ANC) technology has extensive applications in suppressing sound pollution in the real-world environment. In this paper, a new adaptive neuro-fuzzy network (ANFN)-based controller is presented and integrated into hybrid active noise control (HANC) systems to improve the robustness and effectiveness of active noise suppression. Specifically, an adaptive neural network is constructed to minimize the mean square error information with respect to the residual noise. Moreover, a fuzzy logic strategy is proposed to address the manual fine-tuning and nonlinearities encountered in a complex environment. Finally, the stability of the proposed control method is proved by using the Lyapunov theorem. Comparative numerical simulations are given to verify the effectiveness and superiority of the proposed method under different noise signals.

## 1. Introduction

Active noise control (ANC) has been utilized in various applications in aerospace, underwater communications, and industrial manufacturing to mitigate low-frequency noise, showcasing its effectiveness in scenarios where passive noise reduction methods cannot perform well [1]. Hence, ANC is also an essential technology for ensuring a safe working enviroment for humans in high-noise environments [2]. In an ANC system, a secondary noise source is used to produce anti-noise with the same amplitude and opposite phase as the primary noise. These two noises combine to cancel each other in the area where the noise control is needed. The primary path is defined as the transmission path from the noise source to the noise control area, and the secondary path is the transmission path from the secondary noise source generated by the ANC system to the noise control area. Linear ANC systems have been effectively applied to cancel noise in air conditioning ducts, handsets, and various other devices. However, the effectiveness and robustness of ANC may be influenced by nonlinearities and delay in the secondary noise path. To address the above challenges, many ANC methods have been proposed, which can be roughly categorized into feedforward (FF) control, feedback (FB) control, and hybrid control. Feedforward control [3] is used for cases where the noise information can be directly measured, while this condition may not be able to be fulfilled in practice. For the case of unmeasured noise sources, the feedback control method [4,5,6] can be used, where residual noise information is adopted to calculate the output to ensure noise suppression. However, using FF or FB control separately may suffer from performance limitation or even divergence due to the phase mismatch. In this line, a hybrid ANC model (HANC) [7,8,9,10] has been proposed, which combines the advantages of the feedforward and feedback control methods to improve the efficiency of ANC systems. Hence, HANC has attracted much attentions in the last decades.

The conventional HANC system [11] uses the same error signal to adjust both the feedforward and feedback controller. To improve the convergence rate of the traditional hybrid HANC system, a variable step-size method was proposed [10]. In recent work [12], the conventional HANC system has been modified with the online modeling technique of the secondary path. Since the two controllers in the conventional hybrid systems are coupled, the simultaneous optimization of both filters is necessary to achieve stability and the satisfactory performance of the control system. This problem was improved in [13] by changing the reference signal used for the feedback controller and the error signal used for the feedforward controller. Moreover, to improve the convergence speed, a variable step-size approach was proposed for the filter in [7], and the decoupling technique [13] was adjusted for the hybrid structure [14]. In [14], a cascading adaptive filter used hybrid ANC structures to attenuate the uncorrelated narrowband noise at the error microphone without degrading the convergence speed. This method separated the error information into two parts by adding an adaptive filter cascading to the feedforward control filter. In [9], the authors improved the performance of the HANC by using the filtered-X normalized least mean square (FxNLMS) algorithm. Z. Luo et al. [15] suggested a solution by combining the FxNLMS method with a selective fixed filter, where a lightweight one-dimensional convolutional neural network is used to select the best pre-trained control filter for each frame of primary noise. A further tailored HANC system was also suggested [16], which comprises three subsystems: a delayed feedforward narrowband, a robust feedback broadband, and a supporting error calculation subsystem. A delayed notch was used in the least mean square (LMS) algorithm for the feedforward subsystem, and its robustness was enhanced by incorporating the robust control into the feedback subsystem, which can eliminate broadband and narrowband mixed noise in the excavator cab. In general, the HANC systems analyzed above achieve a reliable performance for linear environments, and the proposed improvements are mainly based on changes in the structure of the FxLMS adaptive filter and the step-size of the LMS algorithms.

All the traditional HANC systems mentioned above use Finite Impulse Response (FIR) filters based on the least mean square (LMS) algorithm or its advances [11] as the parameter adjustment mechanism to tune the coefficients of the digital filters to minimize the residual noise signal. Although these HANC schemes have been widely used due to their simplicity and low computational complexity, they are mainly effective for linear systems only, and their performance will degrade when applied to nonlinear ANC systems. However, in practical ANC applications, the primary noise transmission path is usually highly nonlinear, and the secondary path has distortions or reference noise arising from the transmission process. This distortion creates disturbances in the high-frequency range, and thus in some cases, ANC systems based on linear FxLMS controllers become unstable [17,18], such that the noise amplitude is larger than the maximum signal amplitude caused by the distortion of the secondary noise source. In addition, information entropy theory has been introduced to quantify the noise suppression capabilities of different controllers. However, the inherent computational complexity and nonlinear noise sensitivity of entropy limit its ability in practical applications [19]. Based on the above analysis, a conventional linear filter is not effective in attenuating the noise when the ANC system suffers from nonlinearities.

In recent years, some efforts have been made to develop advanced ANC systems with the FxLMS algorithm [20,21,22] to promote the real-world application of ANC systems, e.g., the bilinear FxLMS algorithm [23,24,25], Volterra FxLMS algorithm [26], radial basis function networks [27], fuzzy systems [28,29], recurrent neural networks [30], and other neural networks [31], etc. Specifically, some researchers have focused on proposing new mechanisms to adapt nonlinear systems and accelerate the convergence speed to improve the overall control performance. An adaptive neural fuzzy network [32,33,34] was proposed by combining the fuzzy filter and the neural network as a nonlinear filter. The nonlinear mapping capability of the neural network, together with its exceptional learning ability and the excellent logical reasoning of the fuzzy system, has been proved as a promising solution for complex control systems. However, the HANC system with the neural network and fuzzy system has not been fully explored, since the stability of such HANC system is influenced by the coupling between the feedforward and feedback controls, which inspired the current study.

To further improve the effectiveness and robustness of the HANC system, an adaptive neuro-fuzzy network (ANFN) controller, which combines the fuzzy logic system and neural networks, is developed in conjunction with the FxLMS algorithm to tune the learning weights of the nonlinear filters in the HANC system. The ANFN controller consists of five network layers with online tuning parameters to handle the nonlinear dynamics. The output layer contains a feedback component that returns to its input to monitor, recognize, and send out time-varying patterns and self-adaptive adaptive parameters to enhance noise cancellation. This design helps the proposed HANC model to retain stability and improve its performance even in the presence of nonlinear dynamics. Moreover, an adaptive strategy using the FxLMS algorithm is applied to further reduce the complexity of the HANC systems with the ANFN controllers (ANFN-HANC). In addition, the convergence conditions of all the proposed control systems are thoroughly analyzed using the Lyapunov theory. Extensive simulations demonstrate that the proposed ANFN-HANC method can improve the performance of nonlinear ANC systems. The proposed method has three main stages: (1) the FxLMS algorithm is designed to suppress the noise interference of both the primary and secondary paths; (2) an adaptive neural network is constructed to cancel the residual noise; and (3) a fuzzy logic strategy is proposed to address the limitations of the manual fine-tuning and nonlinearity of the traditional methods.

The rest of this paper is organized as follows: Section 2 outlines the basic principle of the HANC system. Section 3 introduces the proposed ANFN control method and analyzes its convergence. Section 4 compares the proposed method with other mainstream ANC methods via numerical simulation. Section 5 draws some conclusions.

## 2. Traditional HANC System

**Figure 1:**
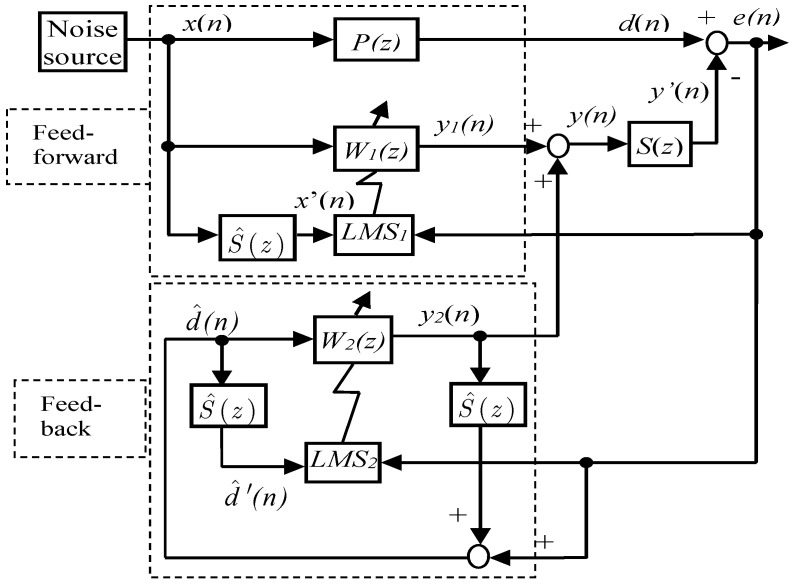
Block diagram of HANC with FxLMS algorithm.

**Figure 2:**
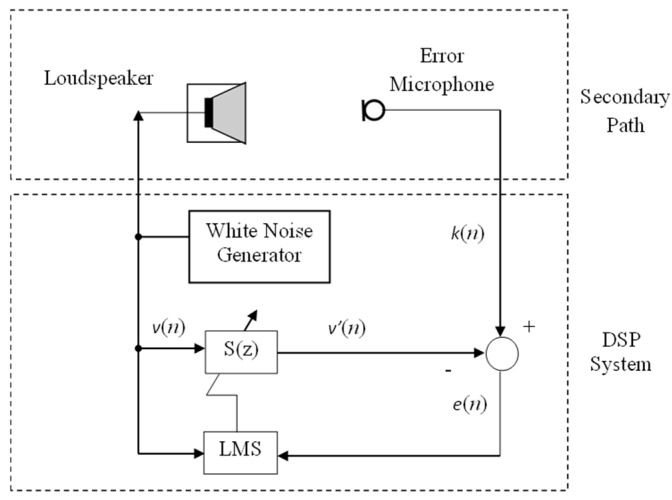
Block diagram of off-line modeling of secondary-path.

The diagram of the traditional HANC system is shown in Figure 1, where P(z) is the primary path from the noise source x(n) to the noise control area d(n), and S(z) is described as the secondary path from the output y(n) of the controller to the secondary noise $y^{\prime }(n)$, which works together with the primary noise d(n) to reduce the error e(n) in the noise control area. The HANC system is the combination of two models, the feedforward model and the feedback model, where $W_{1}(z)$ is the controller of the feedforward model, $W_{2}(z)$ is the controller of the feedback model, and $\hat{S}(z)$ is the estimation of S(z). In practice, $\hat{S}(z)$ can be obtained by using an offline modeling technique [35] without the primary noise, whose idea is given in Figure 2, and the main process involves the following steps:

(a) Generate a sampled white noise signal v(n);
(b) Obtain the desired signal k(n) from the error sensor;
(c) Apply the adaptive filter algorithm: compute the adaptive filter output using an FIR filter and compute the error signal;
(d) Update the coefficients using the LMS algorithm;
(e) Go to step (a) for the next iteration until the adaptive filter S(z) converges and the power of e(n) is minimized.

In Figure 1, the residual noise of the HANC system is given by

(1) $$e(n)=d(n)-y^{\prime }(n)=d(n)-s(n)*y(n)$$

where d(n)=p(n)∗x(n) is the correlated primary noise at the control error area, with ∗ being the convolution operation; p(n) and s(n) are the impulse responses of the primary and secondary paths P(z) and S(z), respectively; and y(n) is the canceling signal, which is the output summation of the controllers $W_{1}(z)$ and $W_{2}(z)$, given by

(2) $$y(n)=y_{1}(n)+y_{2}(n)$$

Moreover, the secondary canceling signal created by the loudspeaker $y^{\prime }(n)$ is given by

(3) $$y^{\prime }(n)=\sum_{j=0}^{J}s_{j}y(n-j)$$

where $s_{j}$ are the coefficients of the $J^{th}$-order FIR filter S(z).

The controllers $W_{1}(z)$ and $W_{2}(z)$ are designed to reduce the total noise e(n) to as small as possible, and the output of $W_{1}(z)$ and $W_{2}(z)$ can be written as

(4) $$y_{1}(n)=\overset{N_{1}-1}{\underset{k_{1}=0}{\sum }}w_{1,k_{1}}(n)x(n-k_{1})=w_{1}^{T}(n)X(n)$$

(5) $$y_{2}(n)=\overset{N_{2}-1}{\underset{k_{2}=0}{\sum }}w_{2,k_{2}}(n)x(n-k_{2})=w_{2}^{T}(n)\hat{d}(n)$$

with

(6) $$w_{1}(n)={\left[w_{1,0}(n),\;w_{1,1}(n),\ldots ,w_{1,N_{1}-1}(n)\right]}^{T}$$

(7) $$w_{2}(n)={\left[w_{2,0}(n),\;w_{2,1}(n),\ldots ,w_{2,N_{2}-1}(n)\right]}^{T}$$

(8) $$X(n)=\left[x(n),\;x(n-1),\ldots ,x(n-N_{1}-1)\right]$$

(9) $$d(n)=\left[d(n),\;d(n-1),\ldots ,d(n-N_{2}-1)\right]$$

where $w_{1}(n),\;w_{2}(n)$ are the parameters of the adaptive filters $W_{1}(z)$, $W_{2}(z)$ with length $N_{1},\;N_{2}$, which are updated online to minimize the synthetic noise using the FxLMS algorithm. $X(n),\;\hat{d}(n)$ are the inputs of the controllers $W_{1}(z),\;W_{2}(z)$. Note, in practice, the secondary path S(z) is usually obtained via offline modeling by $\hat{S}(z)$, so that $s_{j}$ is replaced by its estimate ${\hat{s}}_{j}$. Thus, the estimation of the noise d(n) can be derived by

(10) $$\hat{d}(n)=e(n)+\sum_{j=0}^{J}{\hat{s}}_{j}y_{2}(n-j)$$

with ${\hat{s}}_{j}$ being the coefficients of the $J^{th}$ order of $\hat{S}(z)$, j=0,1,…,J.

To derive the online learning algorithm, we define a mean square cost function as $\xi (n)=E[e^{2}(n)]$, and the weights $w_{1}(n),\;w_{2}(n)$ in the controllers are updated to minimize the error ξ(n) according to the following algorithm:

(11) $$w_{m}(n+1)=w_{m}(n)-\frac{\mu_{m}}{2}\nabla \xi_{m}(n),m=1,2$$

where $\mu_{m}>0$ is the learning gain, and ∇ is the differential operator denoted as

(12) $$\nabla \xi_{m}={\left[\frac{\partial \xi_{m}}{\partial \omega_{m}(0)},\;\frac{\partial \xi_{m}}{\partial \omega_{m}(1)},\ldots ,\frac{\partial \xi_{m}}{\partial \omega_{m}(N_{m}-1)}\right]}^{T}$$

where $N_{m}$ are the order of adaptive digital controllers of the FF model and the FB model, respectively.

The performance and stability conditions of the traditional HANC system depend on the FF and FB models in the system. To simplify the analysis, when the FF model operates independently of the FB model [12], the stability conditions of the HANC system are determined as

(13) $$0<\mu_{1}<2/{\left\|\sum_{j=0}^{J}s_{j}X(n-j)\right\|}^{2}$$

(14) $$0<\mu_{2}<2/{\left\|\sum_{j=0}^{J}s_{j}\hat{d}(n-j)\right\|}^{2}$$

It is noted that the linear FIR filters used in the above controller can ensure stability and convergence. However, its control performance is degraded when applied to nonlinear ANC systems. To overcome the limitations of the above linear HANC methods, this paper will introduce an adaptive neuro-fuzzy network for HANC systems to handle the nonlinear dynamics.

## 3. The Proposed ANFN for the Nonlinear HANC System

In this section, an ANFN is presented to improve the ability to handle the nonlinear dynamics in the HANC systems, which combines both the adaptive neural network and fuzzy logic strategy. The convergence conditions are also derived via the Lyapunov stability theory.

### 3.1. The Proposed ANFN Algorithm

**Figure 3:**
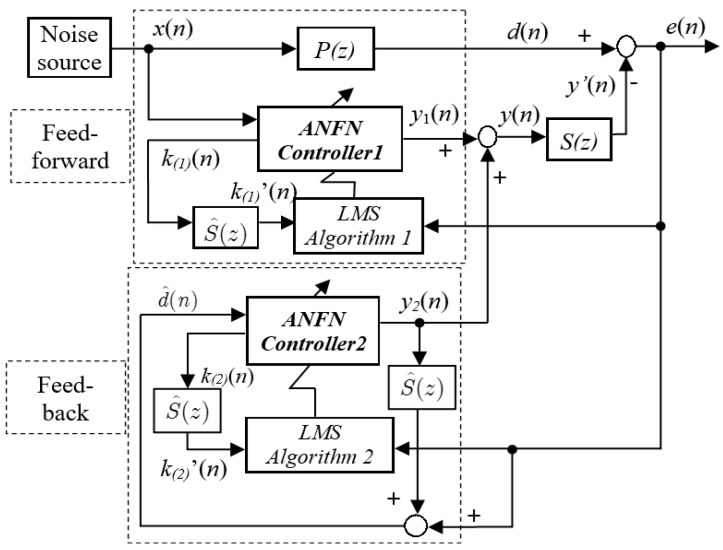
Nonlinear ANFN-HANC system.

The block diagram of the proposed nonlinear HANC system is shown in Figure 3. Compared to the traditional HANC method, it can be seen that the major difference lies in the way that the nonlinear ANFN is designed to replace the traditional linear controllers $W_{m}(z),m=1,2$.

**Figure 4:**
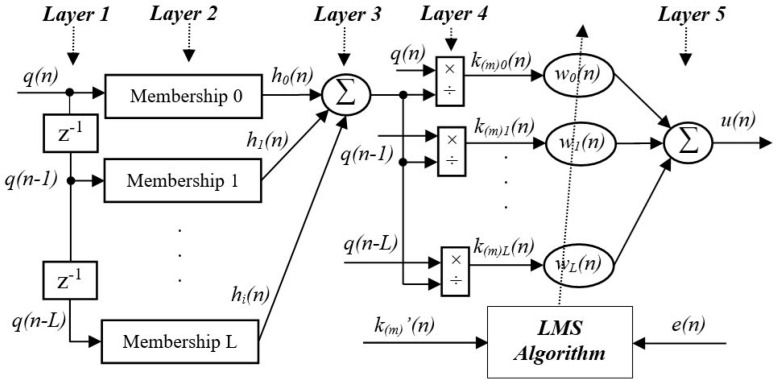
Diagram of adaptive nonlinear ANFN controller.

The specific ANFN controller and the LMS algorithm used to update the weights of the ANFN controller are shown in Figure 4, which can be described by the signal propagation through five layers. The solution for online updating network weights is determined using the FxLMS method, following a nonlinear adaptive mechanism. As shown in Figure 4, denoting $O_{i}^{\left(g\right)}$ as the $i^{th}$ component of layer *g*, the computation of each layer is given as follows:

**Layer 1.**  *Layer 1 is the input layer and the nodes of layer 1 receive the input signal at the $i^{th}$ channel, which directly transmits the input signal to layer 2.*

(15) $$O_{m,i}^{\left(1\right)}(n)=q_{m}(n-i)$$

*where $O_{m,i}^{\left(1\right)}(n)$ is transmitted to layer 2 as the input.*

**Layer 2.**  *In this layer, a membership function with a Gaussian distribution [36] as a fuzzy logic system is designed to handle the nonlinear component as follows:*

(16) $$h_{m,i}=O_{m,i}^{\left(2\right)}=q_{m}(n-i)\cdot a_{m}e^{\frac{-0.5{\left(q_{m}(n)-c_{m}\right)}^{2}}{\sigma_{m}^{2}}}$$

*where $a_{m}$ is a real constant, $c_{m}$ is the mean of the Gaussian function, $\sigma_{m}$ is the width of the Gaussian membership function, and e ≈ 2.718281828 is the Euler constant.*

**Layer 3.**  *This is the summation of Layer 2 as*

(17) $$O_{m}^{\left(3\right)}=\sum_{i=0}^{N_{m}-1}h_{m,i}(n)$$

**Layer 4.**  *In this layer, a normalization operator is introduced to handle the output of the previous layer:*

(18) $$O_{m,i}^{\left(4\right)}=k_{m,i}(n)=\frac{q_{m}(n-i)}{O_{m}^{\left(3\right)}}$$

**Layer 5.**  *This layer is called the output layer. The output of this node integrates all the components in Layer 4 and normalizes the signal by using the FxLMS algorithm to estimate the weights $w_{m,i}(n)$.*

(19) $${O_{m}}^{\left(5\right)}=u_{m}(n)=\sum_{i=0}^{N_{m}-1}w_{m,i}(n)k_{m,i}(n)$$

where $w_{m,i}(n)$ denotes the weight of the neural network, which can be updated online to minimize the total error e(n) of the proposed HANC system by utilizing a novel nonlinear adjustment mechanism.

From Figure 3, we have the residual noise as

(20) $$e(n)=d(n)-y^{\prime }(n)$$

where

(21) $$y^{\prime }(n)=\overset{J}{\underset{j=0}{\sum }}s_{j}y(n-j)$$

with $s_{j}$ being the impulse response of S(z), J the order of S(z), j=0,1,⋯,J.

The anti-noise signal y(n) is the sum of the outputs of the FF model and the FB model, given by

(22) $$y(n)=\overset{2}{\underset{m=1}{\sum }}y_{m}(n),m=1,2$$

As shown in Figure 3, two nonlinear adaptive filters are employed to achieve noise-canceling performance: where the FF ANFN filter (ANFN controller 1) is used to remove the primary noise d(n), and the FB ANFN filter (ANFN controller 2) is used to control the post-processing of the error signal of the FF model using the estimated signal of the reference signal x(n) in the FB model. Defining the mean square cost function as $\xi (n)=E[e^{2}(n)]$, the neural network weights are updated based on the stochastic steepest descent, which incrementally reduces the instantaneous squared error in the output of the ANFN as

(23) $$w_{m}(n+1)=w_{m}(n)-\frac{\mu_{m}}{2}\nabla \xi_{m}(n),m=1,2$$

where $\mu_{m}>0$ is the learning gain, and ∇ is the differential operator of the multivariable function defined by

(24) $$\nabla \xi_{m}={\left[\frac{\partial \xi_{m}}{\partial w_{m}(0)},\frac{\partial \xi_{m}}{\partial w_{m}(1)},\ldots ,\frac{\partial \xi_{m}}{\partial w_{m}(N_{m}-1)}\right]}^{T}$$

where $N_{m}$ are the order of the ANFN controllers of the FF model and the FB model, respectively.

Since the noise d(n) is independent of the weights $w_{m}(n)$, then based on Equation (24), we have

(25) $$\frac{\partial \xi (n)}{\partial w_{m}(n)}=\frac{\partial \xi (n)}{\partial e(n)}\cdot \frac{\partial e(n)}{\partial y^{\prime }(n)}\cdot \overset{J}{\underset{j=0}{\sum }}\left(\frac{\partial y^{\prime }(n)}{\partial y_{m}(n-j)}\cdot \frac{\partial y_{m}(n-j)}{\partial w_{m}(n)}\right)$$

where

(26) $$\begin{array}{l} \frac{\partial \xi (n)}{\partial e(n)}=2e(n),\;\;\;\frac{\partial e(n)}{\partial y^{\prime }(n)}=-1,\;\;\;\;\frac{\partial y^{\prime }(n)}{\partial y_{m}(n-j)}=s_{j},\\ \frac{\partial y_{m}(n-j)}{\partial w_{m}(n)}=k_{m}(n) \end{array}$$

From (25) and (26), we have

(27) $${\left[\frac{\partial \xi (n)}{\partial w_{m}(n)}\right]}^{T}=-e(n)\sum_{j=0}^{J}s_{j}k_{m}(n-j)$$

where $k_{m}(n)$ is the outputs of layer 4 of the ANFN controller 1 and 2, i.e., $k_{m}(n)={\left[k_{m,0}(n),k_{m,1}(n-1),\ldots ,k_{m,N_{m}-1}(n-j)\right]}^{T}$ with length $N_{m}$.

Substituting (27) into (23)–(24), the weights $w_{m}(n)$ are updated by

(28) $$w_{m}(n+1)=w_{m}(n)+\mu_{m}e(n)\sum_{j=0}^{J}s_{j}k_{m}(n-j)$$

The adaptive law (28) is derived based on the FxLMS algorithm; thus the stability of the proposed ANFN-based ANC system should be studied.

### 3.2. Convergence Analysis

The main results can be summarized as follows:

**Theorem 1.**  *For the proposed HANC system shown in Figure 3, with the proposed ANFN given in Figure 4 and the adaptive law (28), if the learning gains fulfill the following conditions, then the proposed HANC system is stable.*

(29) $$0<\mu_{1}<2/{\left\|\sum_{j=0}^{J}s_{j}k_{1}(n-j)\right\|}^{2}$$

(30) $$0<\mu_{2}<2/{\left\|\sum_{j=0}^{J}s_{j}k_{2}(n-j)\right\|}^{2}$$

**Proof.**  The Lyapunov function is defined as

(31) $$V_{m}(n)=\frac{1}{2}e^{2}(n)=\frac{1}{2}{\left[d(n)-y^{\prime }(n)\right]}^{2}$$

Then, the difference in the Lyapunov function (31) can be derived as follows:

(32) $$\begin{array}{l} \Delta V_{m}(n)=V_{m}(n+1)-V_{m}(n)=\frac{1}{2}\left[e^{2}(n+1)-e^{2}(n)\right]\\ \;\;\;\;\;\;\;\;\;\;\;=\frac{1}{2}\left[e(n+1)-e(n)\right]\left[e(n+1)+e(n)\right]\\ \;\;\;\;\;\;\;\;\;\;\;=\frac{1}{2}\Delta e(n)\left[2e(n)+\Delta e(n)\right] \end{array}$$

where Δe(n)=e(n+1)−e(n).

The error Δe(n) is used to update the coefficients $w_{m}(n)$, which can be further analyzed as

(33) $$\begin{array}{l} \Delta e(n)={\left[\frac{\partial e}{\partial w_{m}}\right]}^{T}\Delta w_{m}(n)\\ \;\;\;\;\;\;\;\;={\left[\frac{\partial e(n)}{\partial y^{\prime }(n)}\cdot \overset{J}{\underset{j=0}{\sum }}\left(\frac{\partial y^{\prime }(n)}{\partial y_{m}(n-j)}\cdot \frac{\partial y_{m}(n-j)}{\partial w_{m}(n)}\right)\right]}^{T}\cdot \left[w_{m}(n+1)-w_{m}(n)\right]\\ \;\;\;\;\;\;\;\;=-\mu_{m}e(n){\left\|\overset{J}{\underset{j=0}{\sum }}s_{j}k_{m}(n-j)\right\|}^{2} \end{array}$$

Then, from (32) and (33), we obtain

(34) $$\begin{array}{l} \Delta V_{m}(n)=-\frac{1}{2}\mu_{m}e(n){\left\|\overset{J}{\underset{j=0}{\sum }}s_{j}k_{m}(n-j)\right\|}^{2}\cdot \left(2e(n)-\mu_{m}e(n){\left\|\overset{J}{\underset{j=0}{\sum }}s_{j}k_{m}(n-j)\right\|}^{2}\right)\\ =-\frac{1}{2}\mu_{m}e^{2}(n){\left\|\overset{J}{\underset{j=0}{\sum }}s_{j}k_{m}(n-j)\right\|}^{2}\cdot \left(2-\mu_{m}{\left\|\overset{J}{\underset{j=0}{\sum }}s_{j}k_{m}(n-j)\right\|}^{2}\right) \end{array}$$

From (31), it is shown that $V_{m}(n)\geq 0$ holds and $V_{m}(n)=0$ if and only when e(n)=0 is true. Then, according to the Lyapunov theorem, $V_{m}(n)$ will decrease to zero if the following inequality is satisfied:

(35) $$2-\mu_{m}{\left\|\sum_{j=0}^{J}s_{j}k_{m}(n-j)\right\|}^{2}>0$$

where $k_{m}(n),\;m=1,2$ are the signals extracted from layer 4 of the ANFN controllers. From (15)~(18), we can obtain $k_{m}(n)$ as follows

(36) $$k_{m}(n)=\frac{q_{m}(n)}{\sum_{i=0}^{N_{m}-1}q_{m}(n-i)\cdot a_{m}e^{\frac{-0.5{\left(q_{m}(n)-c_{m}\right)}^{2}}{\sigma_{m}^{2}}}}$$

From Figure 3 and Figure 4, and (36), we have

(37) $$k_{1}(n)=\frac{x(n)}{\sum_{i=0}^{N_{m}-1}x(n-i)\cdot a_{1}e^{\frac{-0.5{\left(x(n)-c_{1}\right)}^{2}}{\sigma_{1}^{2}}}}$$

where $k_{1}(n)$ is the signal extracted from layer 4 of the ANFN controller 1 in the FF ANC model, and $x(n)=q_{1}(n)$ is the input of the controller 1. And

(38) $$k_{2}(n)=\frac{\hat{d}(n)}{\sum_{i=0}^{N_{m}-1}\hat{d}(n-i)\cdot a_{2}e^{\frac{-0.5{\left(\hat{d}(n)-c_{2}\right)}^{2}}{\sigma_{2}^{2}}}}$$

with $k_{2}(n)$ is the signal extracted from layer 4 of the ANFN controller 2 in the FB ANC model, and $\hat{d}(n)=q_{2}(n)$ is the input of the controller 2. □

Consequently, we obtain the conditions to ensure the proposed HANC system’s stability, including the FF and FB models that ensure stability:

(39) $$0<\mu_{1}<2/{\left\|\frac{\sum_{j=0}^{J}s_{j}X(n-j)}{\sum_{i=0}^{N_{m}-1}x(n-i)\cdot a_{1}e^{\frac{-0.5{\left(x(n)-c_{1}\right)}^{2}}{\sigma_{1}^{2}}}}\right\|}^{2}$$

(40) $$0<\mu_{2}<2/{\left\|\frac{\sum_{j=0}^{J}s_{j}\hat{d}(n-j)}{\sum_{i=0}^{N_{m}-1}x(n-i)\cdot a_{2}e^{\frac{-0.5{\left(\hat{d}(n)-c_{2}\right)}^{2}}{\sigma_{2}^{2}}}}\right\|}^{2}$$

which are the detailed versions of those given in (29)–(30).

As shown in Theorem 1, the stability of the proposed HANC system and the convergence of the proposed learning algorithm can be ensured by properly setting the learning gains $\mu_{m}$. Nevertheless, compared with the conventional ANC system given in Section 2, the ANFN algorithm combining the fuzzy logic system and neural networks is used, where the weights are updated to minimize the residual noise signal based on the FxLMS algorithm. In this case, it can handle the spatially incoherent turbulence noise for a HANC system with nonlinearities, so as to achieve a better noise reduction performance. Comparative simulations will be given to verify the above analysis.

### 3.3. Analysis of Computational Complexities

In this subsection, the practical implementation of the proposed controllers will be analyzed, and their computational complexities will be compared with the traditional HANC using the FxLMS algorithm. For simplicity, let $N_{1}=N_{2}=N$.

**(1)**  **Traditional HANC system**

The traditional HANC method uses W(z) as an adaptive linear filter. The implementation of the FIR filter controller can be described as follows:

- Calculate the FIR filter output as $y(n)=\sum_{k=0}^{N-1}w_{k}(n)x^{\prime }(n-k)$, which requires N multiplications and N−1 additions. N denotes the length of the adaptive FIR filter;
- Update the filter weights as w(n+1)=w(n)+μe(n)X(n), which requires N+1 multiplications and N additions;
- Estimating the reference signal as $\hat{d}(n)=e(n)+\overset{J}{\underset{j=0}{\sum }}s_{j}y_{1}(n-j)$ in the feedback model requires J additions and J+1 multiplications.

Therefore, the traditional linear filter-based HANC system needs 4N+J+3 multiplications and 4N+J−1 additions.

**(2)**  **The FF ANC system using the ANFN controller**

This is a specific structure of the proposed HANC, where only the FF model is used, while the FB model is switched off. The computational analysis is given as follows:

- Calculate the output layer 5 of the ANFN controller as $y(n)=\sum_{l=0}^{N-1}w_{l}k_{l}(n)$, which requires N multiplications and N−1 additions, where N is the number of Fuzzy filters used in the ANFN controller;
- Compute layer 4 of the controller as $k_{i}(n)=x(n-i)/O^{\left(3\right)}$, which requires N divisions;
- Calculate the output of layer 3 as $O^{\left(3\right)}=\sum_{l=0}^{N-1}h_{l}(n)$, which requires N−1 additions;
- Compute the $i^{th}$ component of the filter in layer 2 as $O_{i}^{\left(2\right)}=a\cdot x(n-i)e^{\frac{-0.5{\left(x(n)-c\right)}^{2}}{\sigma^{2}}}$, which requires 1 subtraction, 1 division, 5 multiplications, and 1 exponent. Thus, the number of operations in layer 2 is N subtractions, 2N squares, N divisions, 3N multiplications, and N exponentiations;
- Update the weights by $w_{1}(n+1)=w_{1}(n)+\mu_{1}e(n)k_{1}(n)$, which requires N+1 multiplications and N additions.

Therefore, the FF-ANC system with the ANFN controller using the FxLMS algorithm needs 7N+2 multiplications, 2N division, N subtractions, 3N−1 additions, and N exponentiations.

**(3)**  **The FB ANC system using the ANFN controller**

This is another specific structure of the proposed HANC, where only the FB model is used, while the FF model is switched off. The implementation of the ANFN FF model requires computing the signal estimation as follows:

(41) $$\hat{d}(n)=e(n)+\overset{J}{\underset{j=0}{\sum }}s_{j}y_{2}(n-j)$$

which requires J additions and J+1 multiplications.

Therefore, the ANFN-FB controller needs 7N+J+2 multiplications, 2N division, N subtractions, 3N+J−1 additions, and N exponentiations.

**(4)**  **The proposed HANC system**

The implementation of the proposed HANC requires computing two models, the FF-ANC and the FB-ANC, both using the ANFN controller and estimating the reference signal $\hat{d}(n)$ of the FB model. Therefore, the computational load of the proposed HANC system is the sum of the calculations for the ANFN-FF and the ANFN-FB. Therefore, the proposed HANC system needs 14N+J+4 multiplications, 4N division, 2N subtractions, 6N+J−2 additions, and 2N exponentiations.

The comparisons of computational costs of these different four ANC algorithms are summarized in Table 1. It is shown that the proposed HANC method with two ANFN controllers requires more computational costs, which stems from the use of both FF and FB models and the ANFN, aiming at handling the nonlinearities in the system and noises. This increased computational cost is paid to achieve an enhanced control response, and it is feasible for implementation once high-performance digital signal processing (DSP) is used.

## 4. Simulations

In this section, comparative simulation results will be provided to exemplify the superiority of the proposed method over the several traditional ANC methods. In the following simulations, the sampling frequency is set to 8 kHz. The secondary path model S(z) emulating the microphone dynamics is selected as [37]

(42) $$y^{\prime }(n)=y(n-2)+1.5y(n-3)+y(n-4)$$

The order of the adaptive FIR filter is selected as 16, and the learning gain $\mu_{m}$ of the adaptive FIR filter and the ANFN controller are selected as $\mu =\mu_{1}=\mu_{2}=0.001$. Considering the distribution function of the inputs, we set the width c and the means $\delta^{2}$ of the Gaussian membership functions as the same values for various inputs. The fuzzy logic system uses nine fuzzy sets, where c and $\delta^{2}$ of the Gaussian membership functions are determined by a trial-and-error method as

(43) $$\begin{array}{c} \;\;\;\;c=\left[-1,-0.75,-0.5,-0.25,0,0.25,0.5,075,1\right]\\ \delta^{2}=\left[0.4,0.2,0.2,0.2,0.2,0.2,0.2,0.2,0.4\right]\;\;\;\;\; \end{array}$$

**Figure 5:**
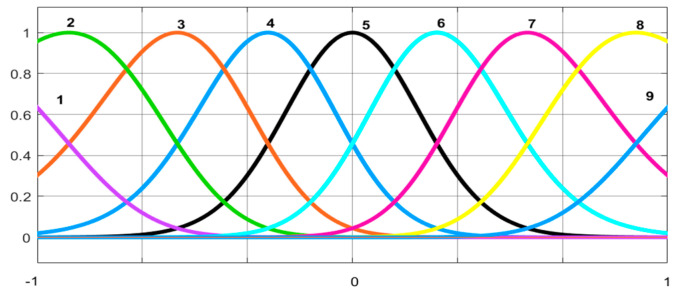
An initial membership function setting in our simulation.

The graphical representation of the means and widths of the Gaussian membership is given in Figure 5. The operating range is set as [0,1]. When the HANC system is operating, the widths c and means $\delta^{2}$ of the Gaussian membership functions are fixed, and only the weight vectors $w_{m}(n)$ are adaptively adjusted online.

**Case 1.**  *In this case, we choose as a noise source a sinusoidal signal with a frequency of 30 Hz, and a nonlinear primary path that is a nonlinear second-order polynomial, as in [31], is used*

(44) $$d(n)=x(n-3)-0.3x(n-4)+0.8x^{2}(n-4)+0.2x(n-5)$$

To illustrate the effectiveness of the proposed ANFN controllers, we compare the proposed HANC including the ANFN controller (ANFN- HANC) with the traditional linear FIR filter-based control (4) and (5) with the FxLMS algorithm (11) (FxLMS) and the artificial neural network controller (ANN-Thai model) proposed in [31], where the input of the ANN controller is chosen as N=32 and the learning gains $\mu =\mu_{1}=\mu_{2}=0.001$.

**Figure 6:**
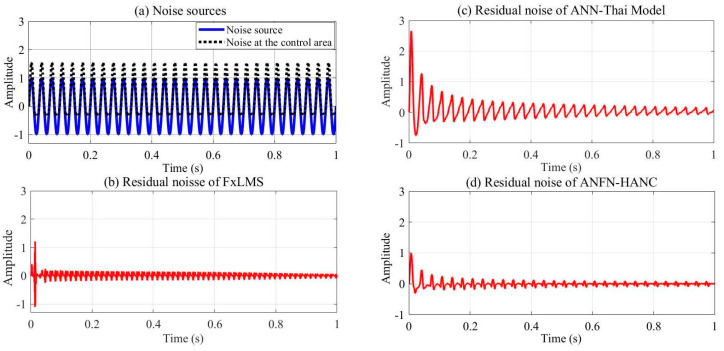
Simulation results for 30 Hz noise in time domain.

**Figure 7:**
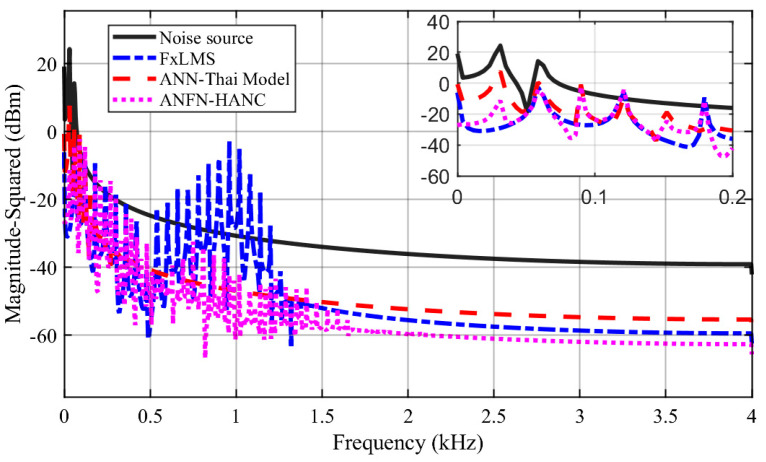
Simulation results for 30 Hz noise in frequency domain.

**Figure 8:**
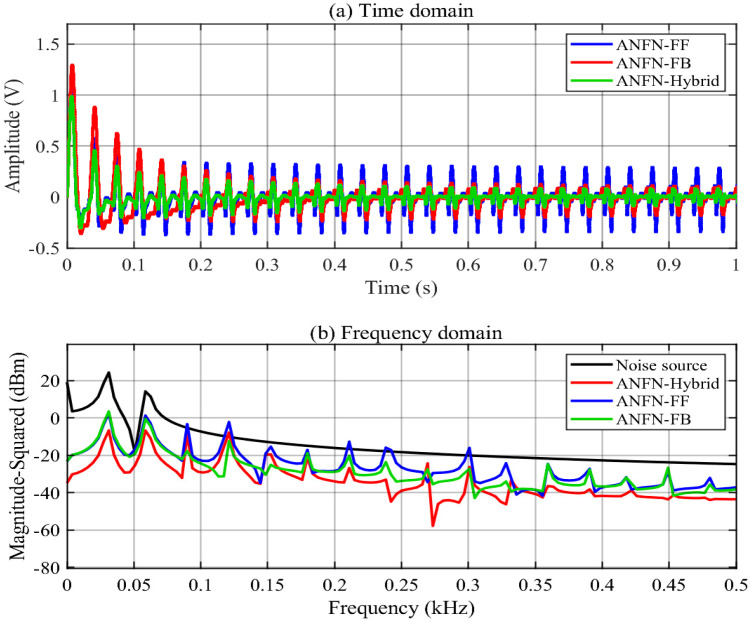
Simulation results for 30 Hz noise with different ANFN structures.

The simulation results of these ANC systems are shown in Figure 6, Figure 7 and Figure 8. Figure 6 depicts the noise source signals and the control performance of these three different approaches in the time domain. Figure 6a shows the noise source signals and the noise after transmission through the nonlinear primary path. The blue line displays the noise source of the ANC system, and the dotted black line depicts the noise at the control area. This figure shows that the noise in the control area changes in amplitude compared to the original noise due to the nonlinearities in the primary path. These nonlinear dynamics will reduce the performance or can even lead to the system deviation and instability of ANC systems (because the reference signal taken from the noise source as the reference signal for the controller is different from the output signal of the controller). Figure 6b,c show the residual noise signal of the FIR-based FxLMS method and the ANN-Thai model [31] in the time domain, Figure 6c shows the control performance of the proposed ANFN-HANC in the time domain. Figure 7 shows the simulation results of three controllers in the frequency domain, where the dash–dot dark blue line, dashed red line, and dotted purple line represent the power spectrum of the residual noise for the FIR-based FxLMS method, the ANN-Thai model, and the proposed ANFN-HANC system, respectively. In Figure 7, the FIR-based FxLMS method does not achieve convergence in the specific frequency around 1 kHz due to the nonlinearities in the transmission path of the ANC system [17]. From these figures, we can see that the performance of the proposed ANFN-HANC system using the ANFN controllers is more effective than the other two control methods.

To further verify the necessity of using the hybrid scheme with both the FF and FB models, we also provide comparative simulations with the proposed ANFN-HANC and its two simplifications with only the FF model or FB model given in Section 3.3. Figure 8a illustrates the residual noise at the control area of these three different structures for the proposed ANFN controller. The solid blue line, solid red line, and solid green line represent the residual noise of the feedforward ANC, the feedback ANC, and the proposed HANC, respectively. This figure demonstrates that the performance of the FB model is better than the FF model (because the reference signal fluctuates too much compared to the signal in the noise control area), and the performance of the proposed ANFN-HANC is the best. Figure 8b displays the spectrum of the signals of three cases, FF, FB, and the proposed ANFN-HANC, in the frequency domain. The solid black line shows the spectrum of the noise source. The solid blue, solid green, and solid red lines indicate the spectrum of the residual noise for the proposed control with FF model, the FB model, and the proposed ANFN-HANC, respectively. This figure also shows that the proposed ANFN-HANC can achieve the best control performance.

**Case 2.**  *In this case, we use a sinusoidal noise with two frequencies: 50 Hz and 100 Hz. All other parameters for the control systems are selected the same as Case 1.*

**Figure 9:**
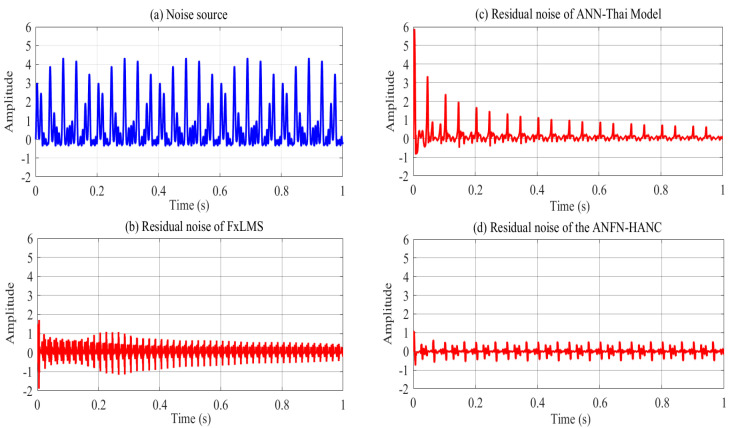
Simulation results for noise with frequencies of 50 Hz and 100 Hz in time domain.

**Figure 10:**
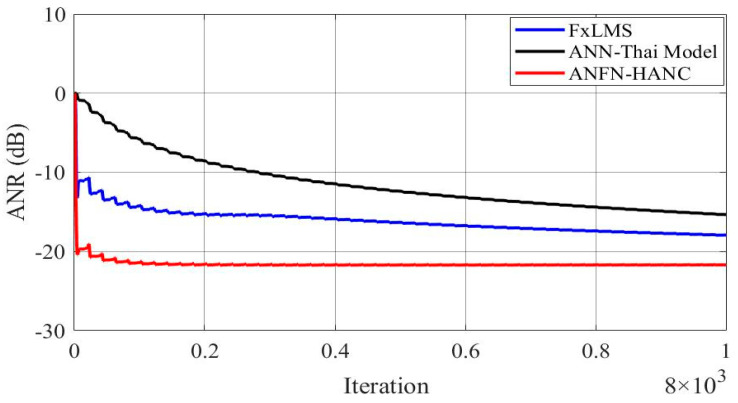
ANR of residual noise with different ANC methods at frequencies of 50 Hz and 100 Hz.

Figure 9 presents the simulation results in the time domain. Figure 9a shows the noise at the control area, and Figure 9b–d display the residual noise of three different control systems in the time domain: the FIR-based FxLMS method, the ANN-Thai model-based method proposed in [31], and the proposed ANFN-HANC method. Moreover, the statistical performance indices are also used to evaluate the control performance, which is shown in Table 2: Mean Absolute Error ($\mathrm{MAE}=\frac{1}{N_{s}+1}\overset{N_{s}}{\underset{n=0}{\sum }}|e(n)|$), mean square error ($\mathrm{MSE}=\frac{1}{N_{s}+1}\overset{N_{s}}{\underset{n=0}{\sum }}e^{2}(n)$) and Standard Deviation ($\mathrm{SD}=\sqrt{\frac{1}{N_{s}}\overset{N_{s}}{\underset{n=0}{\sum }}(|e(n)|-\mathrm{MAE})}$). For Case 2, the control error performance of the ANFN-HANC shows significant improvements compared to the other methods. Specifically, compared to the FIR-HANC, the proposed ANFN-HANC achieves a reduction of 33% in MAE, 44% in MSE, and 23% in SD. As compared to the ANN-Thai scheme, the proposed ANFN-HANC reduces the MAE by 46%, MSE by 86%, and SD by 60%. These results highlight the superior performance of the ANFN-HANC in Case 2. Furthermore, to evaluate the effectiveness of the methods, the average noise reduction ($ANR(dB)=10\log_{10}\left\{\frac{E\left[e^{2}(n)\right]}{E\left[d^{2}(n)+{y^{\prime }}^{2}(n)\right]}\right\}$) is shown in Figure 10. In this case, the proposed HANC system has the lowest steady-state ANR, followed by the HANC system using the adaptive FIR filter, and the ANN-Thai model. Among the three methods, the proposed HANC with the ANFN controller stands out with a significantly improved steady-state performance compared to the other two methods.

**Figure 11:**
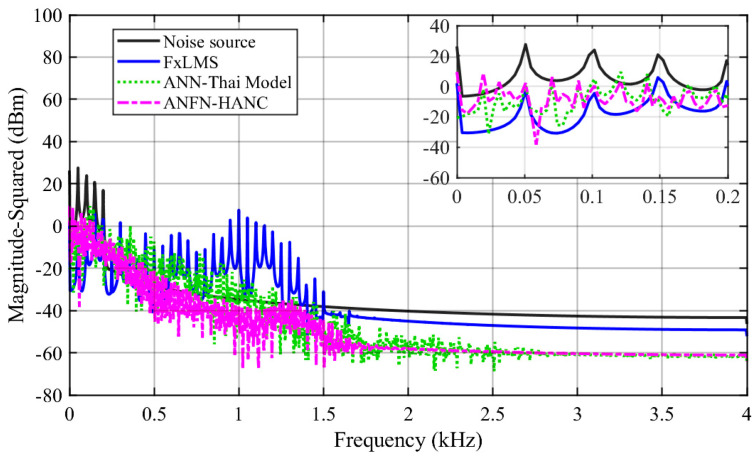
Simulation results for noise with frequencies of 50 Hz and 100 Hz in frequency domain.

Figure 11 displays the spectrum of the signals of three ANC systems in the frequency domain, where the spectrum of the noise source is shown in the solid black line. The solid blue, dotted green, and dash–dot purple lines indicate the spectrum of the residual noise for the FIR-based FxLMS method, the ANN-Thai model-based method proposed in [31], and the proposed ANFN-HANC method, respectively. From these figures, we can see that the linear FIR filter with FxLMS responds to reduce the residual noise in a specific frequency band, but for the frequency band outside the noise control range, it shows an increased trend, which may lead to instability of the FIR filter. This can be explained as follows: when adding two signals together, in addition to the center frequency signals with the largest amplitude, there are signals with a small amplitude other than the center frequency, and these frequencies (which can be considered as nonlinearity in the system) make the linear FIR filter unstable [17,18], which will also be shown in Case 3. It can also be observed from these figures that the performance of the proposed ANFN-HANC system is more effective than the traditional FIR-based FxLMS method and ANN-Thai model-based method.

**Figure 12:**
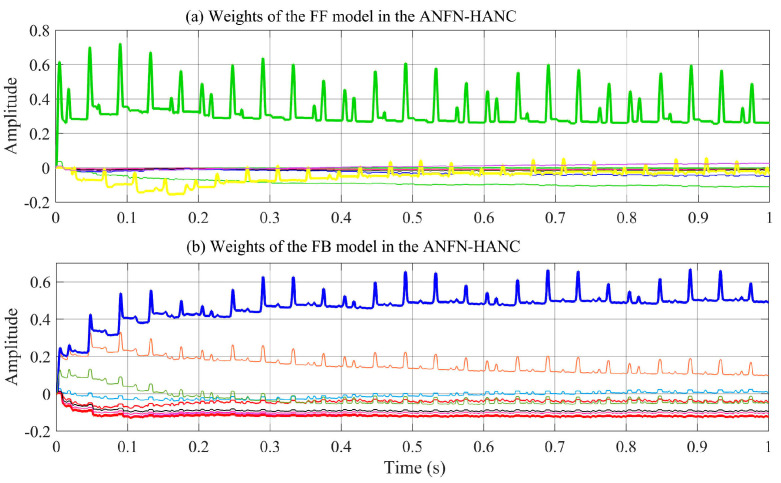
Weights of the FF, FB model used for the proposed ANFN-HANC system.

Figure 12 displays the profile of the NN weights $w_{m}(n)$ with the proposed adaptive law (28) for the FF and FB controllers. From this figure, we can see that the coefficients $w_{m}(n)$ do not converge to fixed values around a fixed point, but they remain bounded and show convergent trends. In fact, it can be found from (28) that the coefficients $w_{m}(n)$ converge to constant values if and only if e(n)=0.

**Case 3.**  *To confirm the reliability of the proposed ANFN-HANC system with strong nonlinearities, we further use a multi-tonal noise signal consisting of frequencies of 50 Hz, 100 Hz, and 120 Hz. Moreover, the primary path is selected as a third-order nonlinear polynomial as follows:*

(45) $$d(n)=x(n-3)-0.3x(n-4)+0.8x^{3}(n-4)+0.2x(n-5)$$

**Figure 13:**
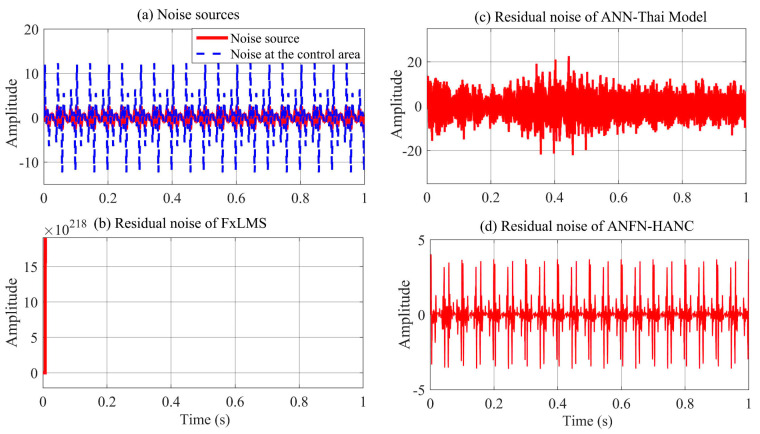
Simulation results noise with frequencies of 50 Hz, 100 Hz, and 120 Hz in time domain.

**Figure 14:**
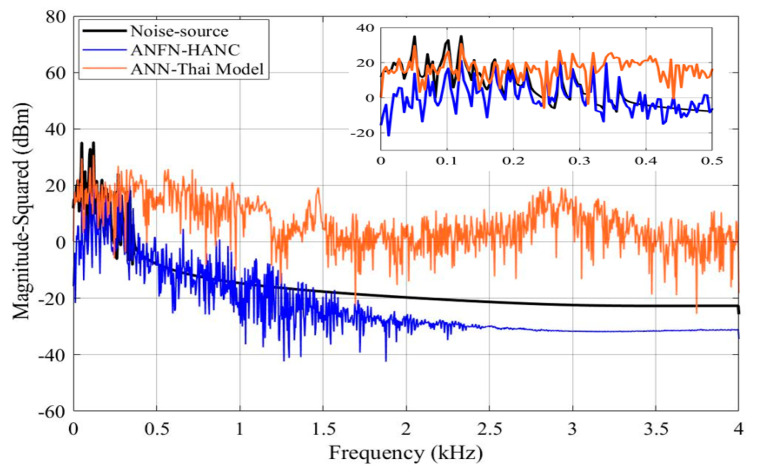
Simulation results for noise with frequencies of 50 Hz, 100 Hz, and 120 Hz in frequency domain.

**Figure 15:**
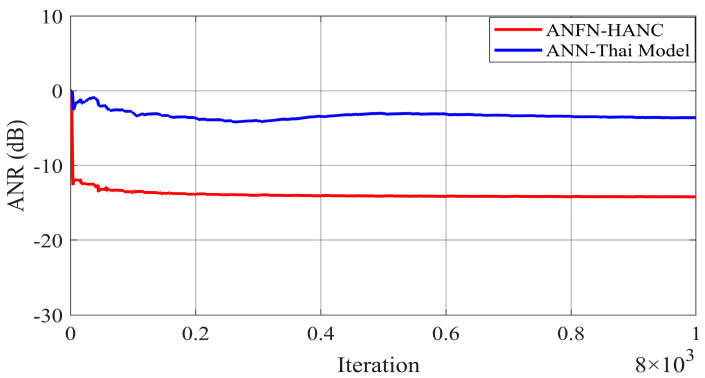
ANR of residual noise with different ANC methods at frequencies of 50 Hz, 100 Hz, and 120 Hz.

All other parameters of the ANC systems are selected as the same as Case 2. Comparative simulation results are shown in Figure 13, Figure 14 and Figure 15. Figure 13a presents the noise source and noise signal in the control area in the time domain, which indicates that the noise in the control area changes significantly compared to the original noise. The nonlinear dynamics will reduce the performance of the ANC systems. Figure 13b illustrates the control response of the traditional FIR-based FxLMS HANC system using the linear FIR filter in the time domain, which triggers instability as in [17,18]. Figure 13c,d display the residual noise signal of the ANN-Thai model-based system and the proposed ANFN-HANC system. These figures also show that the proposed ANFN-based controllers can retain stability and achieve a fairly small residual noise better than the ANN-Thai model-based ANC system. To be specific, from the quantitative error indices given in Table 3, it is found that, compared to the FIR-HANC, the control error performance of ANFN-HANC achieves a reduction of 86% in MAE, 96% in MSE, and 81% in SD. Figure 14 shows the spectrum signal of these three methods in the frequency domain. Since the FIR-based ANC system with the FxLMS method cannot retain stability due to the nonlinearities in the transmission path, its spectrum is not shown in Figure 14. These results also support the theoretical analysis given in Section 4. Figure 15 shows the ANR of the proposed ANFN HANC system with the red colored line and the ANR of the ANN-Thai model with the blue colored line. This figure also demonstrates that the proposed ANFN-HANC has a more reliable noise reduction capability.

**Figure 16:**
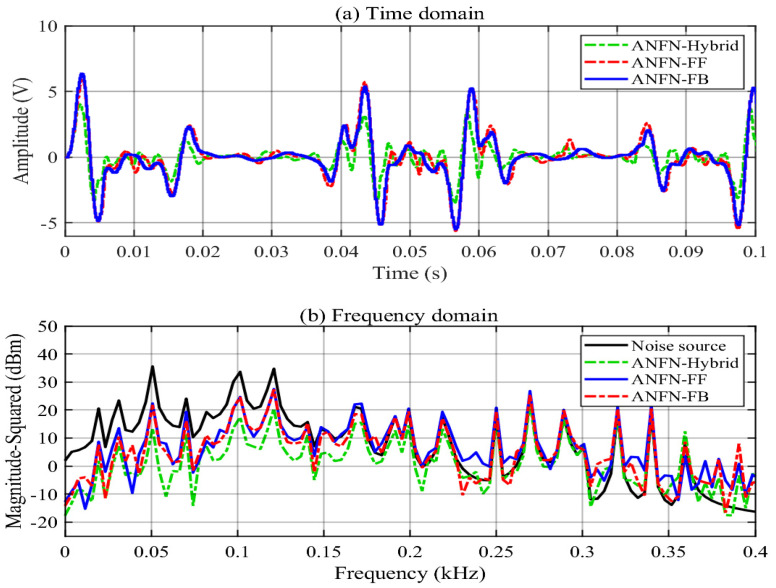
Simulation results for noise with frequencies of 50 Hz, 100 Hz, and 120 Hz of ANFN methods.

Finally, the use of a hybrid structure with both FF and FB controllers is validated. Figure 16a shows the residual noise of the proposed HANC systems with different control configurations in the time domain, i.e., the feedforward ANC, the feedback ANC, and the proposed ANFN-HANC, while Figure 16b presents the spectrum signal of the residual noise in the frequency domain. From these figures, we can see that the performance of the feedback model is slightly better than the feedforward model, which is similar to Case 1. Moreover, the proposed ANFN-HANC system with both feedback and feedforward controllers obtains the best performance.

In the above three simulation studies, the proposed ANFN-HANC system can achieve the best noise control responses. Compared to the other investigated ANC systems, it also shows the best performance in terms of robustness, noise suppression, and convergence rate.

## 5. Conclusions

In this paper, we present a nonlinear hybrid control design for ANC systems using two ANFN controllers as an alternative to adaptive FIR filters. The proposed ANFN-based hybrid ANC system, which combines both the feedforward and feedback models, uses a novel nonlinear parameter adjustment mechanism to adjust the coefficients of neural networks to handle nonlinearities in the ANC systems. This approach minimizes the residual noise signal, accelerates the convergence speed, and improves the overall control performance. In particular, we have performed a comprehensive analysis of the convergence conditions of the nonlinear ANFN-HANC systems. Finally, comparisons between the traditional FIR filter-based ANC system with the FxLMS algorithm and the proposed ANFN-HANC systems, with different simplifications in terms of computational complexity, are also given. The results of extensive comparative numerical simulations with different error information indices also show the efficiency of the proposed HANC system with ANFN controllers over some existing methods.

## Figures and Tables

**Table 1:** Comparisons of computational costs.

Controllers	+	−	×	÷	exp(⋅)
FIR-HANC	4N+J−1	0	4N+J+3	0	0
ANFN-FF	3N−1	N	7N+2	2N	N
ANFN-FB	3N+J−1	N	7N+J+2	2N	N
ANFN-HANC	6N+J−2	2N	14N+J+4	4N	2N

**Table 2:** Quantitative comparison of different HANC methods in Case 2.

		FIR-HANC	ANN-Thai	ANFN-HANC
Case 2	MAE	0.159	0.197	0.107
	MSE	0.056	0.216	0.031
	SD	0.230	0.442	0.176

**Table 3:** Quantitative comparison of different HANC methods in Case 3.

		FIR-HANC	ANN-Thai	ANFN-HANC
Case 3	MAE	—	4.446	0.601
	MSE	—	28.596	1.042
	SD	—	5.348	1.021

## Data Availability

The data presented in this study are available on request from the corresponding author.
